# *Escherichia*/*Shigella*, SCFAs, and Metabolic Pathways—The Triad That Orchestrates Intestinal Dysbiosis in Patients with Decompensated Alcoholic Cirrhosis from Western Mexico

**DOI:** 10.3390/microorganisms10061231

**Published:** 2022-06-16

**Authors:** Tonatiuh Abimael Baltazar-Díaz, Luz Alicia González-Hernández, Juan Manuel Aldana-Ledesma, Marcela Peña-Rodríguez, Alejandra Natali Vega-Magaña, Adelaida Sara Minia Zepeda-Morales, Rocío Ivette López-Roa, Susana del Toro-Arreola, Erika Martínez-López, Adriana María Salazar-Montes, Miriam Ruth Bueno-Topete

**Affiliations:** 1 Instituto de Investigación en Enfermedades Crónico-Degenerativas, Departamento de Biología Molecular y Genómica, Centro Universitario de Ciencias de la Salud, Universidad de Guadalajara, Sierra Mojada 950, Guadalajara CP 44340, Jalisco, Mexico; tonatiuhabd@gmail.com (T.A.B.-D.); susana@cucs.udg.mx (S.d.T.-A.); asalazar_montes@hotmail.com (A.M.S.-M.); 2 Instituto de Investigación en Inmunodeficiencias y VIH, Departamento de Clínicas Médicas, Centro Universitario de Ciencias de la Salud, Universidad de Guadalajara, Hospital 278, Guadalajara CP 44280, Jalisco, Mexico; luceroga08@gmail.com; 3 Unidad de VIH, Hospital Civil de Guadalajara, Unidad Hospitalaria Fray Antonio Alcalde, Hospital 278, Guadalajara CP 44280, Jalisco, Mexico; 4 Servicio de Gastroenterología, Hospital Civil de Guadalajara, Unidad Hospitalaria Fray Antonio Alcalde, Hospital 278, Guadalajara CP 44280, Jalisco, Mexico; jumal13@hotmail.com; 5 Laboratorio de Diagnóstico de Enfermedades Emergentes y Reemergentes, Departamento de Microbiología y Patología, Centro Universitario de Ciencias de la Salud, Universidad de Guadalajara, Sierra Mojada 950, Guadalajara CP 44340, Jalisco, Mexico; marcee24.p.r@gmail.com (M.P.-R.); alejandra.vega@academicos.udg.mx (A.N.V.-M.); 6 Instituto de Investigación en Ciencias Biomédicas, Departamento de Clínicas Médicas, Centro Universitario de Ciencias de la Salud, Universidad de Guadalajara, Sierra Mojada 950, Guadalajara CP 44340, Jalisco, Mexico; 7 Laboratorio de Investigación y Desarrollo Farmacéutico, Centro Universitario de Ciencias Exactas e Ingenierías, Universidad de Guadalajara, Blvd. Marcelino García Barragán 1421, Guadalajara CP 44430, Jalisco, Mexico; adelaida.zepeda@academicos.udg.mx (A.S.M.Z.-M.); rocio.lopez@academicos.udg.mx (R.I.L.-R.); 8 Instituto de Nutrigenética y Nutrigenómica Traslacional, Departamento de Biología Molecular y Genómica, Centro Universitario de Ciencias de la Salud, Universidad de Guadalajara, Sierra Mojada 950, Guadalajara CP 44340, Jalisco, Mexico; erikamtz27@yahoo.com.mx

**Keywords:** liver cirrhosis, microbiome, SCFA, *Escherichia*, butyrate, alcohol

## Abstract

Gut microbiota undergoes profound alterations in alcohol cirrhosis. Microbiota-derived products, e.g., short chain fatty acids (SCFA), regulate the homeostasis of the gut-liver axis. The objective was to evaluate the composition and functions of the intestinal microbiota in patients with alcohol-decompensated cirrhosis. Fecal samples of 18 patients and 18 healthy controls (HC) were obtained. Microbial composition was characterized by 16S rRNA amplicon sequencing, SCFA quantification was performed by gas chromatography (GC), and metagenomic predictive profiles were analyzed by PICRUSt2. Gut microbiota in the cirrhosis group revealed a significant increase in the pathogenic/pathobionts genera *Escherichia*/*Shigella* and *Prevotella*, a decrease in beneficial bacteria, such as *Blautia*, *Faecalibacterium*, and a decreased α-diversity (*p* < 0.001) compared to HC. Fecal SCFA concentrations were significantly reduced in the cirrhosis group (*p* < 0.001). PICRUSt2 analysis indicated a decrease in acetyl-CoA fermentation to butyrate, as well as an increase in pathways related to antibiotics resistance, and aromatic amino acid biosynthesis. These metabolic pathways have been poorly described in the progression of alcohol-related decompensated cirrhosis. The gut microbiota of these patients possesses a pathogenic/inflammatory environment; therefore, future strategies to balance intestinal dysbiosis should be implemented. These findings are described for the first time in the population of western Mexico.

## 1. Introduction

In Mexico, liver cirrhosis is the sixth leading cause of death, where alcohol represents one of the main causes of this chronic disease [1]. Cirrhosis is associated with marked alterations of the gut-liver axis, particularly in the advanced stages of the disease; therefore, there is a significant gut microbiota dysbiosis [2].

Dysbiosis occurs when bacteria no longer live in a mutualistic association, within an ecosystem. Characteristic hallmarks of this dysbiosis are the loss of global microbial diversity and, in parallel, excessive growth of species called pathobionts, which are genetic variants of the “pathogenic” microbiota; moreover, the location of microorganisms in the intestinal tract can be modified. Dysbiosis is a disease-associated imbalance that alters the microbiota metabolic products, impacting the host′s immune system [3].

In alcoholic cirrhosis, the gut microbiota experiences significant changes characterized by a depletion of potential beneficial taxa (such as Lachnospiraceae, Ruminococcaceae, and Clostridiales) [2,4,5]; as well as an expansion of the abundance of phyla Proteobacteria and Bacteroidetes. Within these phyla, the most critical overrepresented familiae are Enterococcaceae and Enterobacteriaceae. Consequently, endotoxins derived from these taxa are thought to be an essential trigger for systemic inflammation that may lead to sepsis and death [2,6]. Most of these findings have been reported in American and Asian populations [2,7].

It is relevant to mention that in the Mexican population, very few studies have analyzed the taxonomic features of the intestinal microbiota. The Mexican population has unique characteristics that make it attractive for this type of study, such as a high-fat and high-carbohydrate diet, as well as a genetic predisposition to metabolic diseases [4,8]; consequently, these factors could impact the natural history of cirrhosis.

On the other hand, SCFA (short chain fatty acids) are metabolites derived from the bacterial fermentation of nondigestible polysaccharides, mainly made up of acetate, propionate, and butyrate [9,10]. While acetate is used by the healthy liver for cholesterol and long-chain fatty acids synthesis, propionate serves as a substrate for gluconeogenesis [9,10,11]. Butyrate is the main energy source for colonocytes and has interesting anti-inflammatory and immunomodulatory properties, as well as a demonstrated histone deacetylase inhibitor activity [10,12,13,14,15].

Furthermore, SCFA are important contributors in processes implicated in the pathophysiology of cirrhosis, such as the maintenance of a gut barrier function, immune regulation, anti-inflammatory effects, and the regulation of microbiota itself [16,17,18].

Along with taxonomical alterations, there is a modification in the functional ability of the gut microbiota in patients with cirrhosis, showing a decreased capacity to ferment SCFA [19].

Despite liver cirrhosis currently accounting for a significant number of deaths, the taxonomic profile of gut microbiota in patients from western Mexico is unknown. Additionally, its functional aspects, such as SCFA levels, are much less well known. Therefore, describing these unknown aspects was the central aim of this study. We describe a gut microbiota with a dominant abundance of *Escherichia*/*Shigella*, with a consequent loss of beneficial functions, such as production of SCFA. This is well correlated with diminished metabolic pathways, such as the fermentation of acetyl-CoA to butyrate.

## 2. Materials and Methods

### 2.1. Approval of Clinical Research

This cross-sectional observational study was carried out at the Hospital Civil de Guadalajara, in Guadalajara, Jalisco, Mexico. The study was in accordance with the guidelines of the World Medical Association (Declaration of Helsinki, revised in 2013) and was approved by the Ethics Committee of the named hospital (010/20). The purpose of the study was explained to the subjects and written consent was obtained from the participants.

### 2.2. Study Design

Thirty-six participants were included in the study; 18 male inpatients were recruited from the Gastroenterology Service of the Hospital Civil de Guadalajara Fray Antonio Alcalde, from August 2020 to May 2021. Additionally, 18 healthy controls were recruited from the community.

Inclusion criteria for patients with alcoholic cirrhosis were: (a) Decompensated inpatients with diagnosis of cirrhosis by biopsy, or by clinical criteria with imaging confirmation, within Child–Pugh category B or C. (b) Age between 18 to 70 years, (c) BMI between 18.5 and 29.9 kg/m^2^.

Decompensated alcoholic cirrhosis was defined when patients had 1 or more of the following complications: ascites, jaundice, gastrointestinal bleeding, hepatic encephalopathy and/or bacterial infections. All inpatients were under lactulose or antibiotic prophylaxis.

Excessive consumption of alcohol 72 h before recruiting (>48 g alcohol/day), use of prebiotics/probiotics 4 weeks before recruiting, HIV, hepatitis B or C infection, current or past severe SARS-CoV-2 infection, or any chronic gastrointestinal or autoimmune diseases were non-inclusion criteria.

Inclusion criteria for healthy subjects (control group) were: (a) Age between 18 to 70 years, (b) BMI between 18.5 and 29.9 kg/m^2^, (c) No current or past SARS-CoV-2 infection for at least 3 months before recruiting, (d) No use of prebiotics/probiotics 4 weeks before recruiting, (e) No use of antibiotics 3 months before recruiting, (f) No known allergies or intolerances to fiber sources, (g) Non-vegetarian or smokers, (h) Alcohol intake ≤ 28 g alcohol/week.

### 2.3. Extraction of Nucleic Acids and 16S rRNA Amplicon Sequencing

Fecal samples were collected and immediately stored at −80 °C. DNA was extracted from 250 mg of frozen feces with QIAamp PowerFecal DNA Kit (QIAGEN, Hilden, Germany) according to the manufacturer’s protocol. DNA was quantified with NanoDrop™ One^C^ spectrophotometer (Thermo Scientific, Waltham, MA, USA).

16S metagenomic sequencing library preparation was performed according to Illumina MiSeq System protocol (Illumina, San Diego, CA, USA) [20]. V3 and V4 regions from 16S were amplified with Platinum Taq DNA Polymerase High fidelity (Invitrogen, Waltham, MA, USA) using primers with adaptors. The sequence of the primers used was: Forward: (5′TCGTCGGCAGCGTCAGATGTGTATAAGAGACAGCCTACGGGNGGCWGCAG-3′), reverse: (5′GTCTCGTGGGCTCGGAGATGTGTATAAGAGACAGGACTACHVGGGTATCTAATCC-3′). PCR conditions were followed according to protocol. Product purification was achieved with AMPure XP^®^ (Beckman Coulter, Indianapolis, IN, USA) magnetic beads and was quantified with Qubit^®^ 3 dsDNA HS kit (Invitrogen, Waltham, MA, USA) according to product indications. Next, index incorporation was achieved with Nextera XT Index Kit v2 Set A (No. Cat. FC-131-2001, Illumina, San Diego, CA, USA) by a second PCR amplification. Finally, amplicons were pooled to equimolar concentrations into a 4 nmol/L solution tube, library denaturing and MiSeq Sample Loading (kit Miseq Reagent V3 600-cycle, Illumina, San Diego, CA, USA) according to protocol.

### 2.4. Bioinformatic Analysis of 16S Amplicon Sequencing

Microbiome bioinformatics were performed with QIIME2 version 2021.8 [21]. Raw sequence data quality filtered by denoising with DADA2 via q2-dada2 [22]. All amplicon sequence variants (ASVs) were aligned with MAFFT [23] (via q2-alignment) and used to construct a phylogeny with FastTree2 [24] (via q2-phylogeny). Taxonomy was assigned to ASVs (amplicon sequence variants) using the q2-feature-classifier [25] classify-sklearn naïve Bayes taxonomy classifier against the Silva 138 99% OTUs full-length sequences [26,27]. Alpha diversity metrics (observed features, Shannon and Chao1 indices [28]) were calculated with a QIIME2 pipeline. Beta diversity metrics (Weighted UniFrac and unweighted UniFrac [29,30]), and Principle Coordinate Analysis (PCoA) were generated and tested with Microbiome Analyst [31,32]. Linear discriminant analysis effect size (LEfSe) was obtained with the Galaxy interface [33,34]. The threshold cutoff value of LDA score was 4.0. Phylogenetic Investigation of Communities by Reconstruction of Unobserved States (PICRUSt2) pipeline [35,36,37,38,39] was used to predict the functional pathways of each group according to the MetaCyc Database [40]. ANCOM (analysis of the composition of microbiomes), which is a compositionally based method [41], was performed at the genus level to determine differentially abundant taxa using the q2composition plugin implemented in the QIIME2 pipeline.

### 2.5. Analysis of Fecal SCFAs

SCFA quantification was performed as reported by Ribeiro et al. [42]. Fecal samples frozen at −80 °C were transferred (20 mg) to 200 µL to solution A (N-butanol, tetrahydrofuran and acetonitrile in a 50:30:20 ratio; 40 µL HCl 0.1 M, 20 mg citric acid and 40 mg NaCl), homogenized with vortex for 1 min and centrifuged at 13,000× *g* at room temperature for 10 min. Supernatant was filtered through a 0.22 μm filter (Whatman GD/X, Merck, Darmstadt, Germany) and transferred to chromatographic vials, and 3 µL were injected into the Shimadzu Gas Chromatograph (Shimadzu Scientific Instruments, Kyoto, Japan). SCFAs were separated on a Mega-Acid FFAP column (30 m × 0.25 mm × 0.40 μm, Mega, Milan, Italy) with a flame ionization detector. Concentrations of SCFA in fecal samples were normalized to the wet weight of feces. Total SCFA were determined as the sum of acetate, propionate, and butyrate concentrations.

### 2.6. Statistical Analyses

When assessing the participants’ background characteristics, Student′s *t*-test or Mann-Whitney U test were employed, depending on parametric or non-parametric variables. Alpha diversity metrics among groups were compared using U of Mann-Whitney test, or Kruskal–Wallis with Benjamini, Krieger and Yekutieli multiple testing when assessing subgroups. Beta diversity metrics among groups were compared performing PERMANOVA tests through a MicrobiomeAnalyst server. Benjamini–Hochberg multiple testing corrections were performed when evaluating subgroups through a QIIME2 package. PICRUSt2 outputs were tested using Welch’s inverted method and STAMP software [43]. SCFA differences among groups were assessed using the Mann-Whitney U test. All statistical tests were two-sided, and a *p*-value or false discovery rate-adjusted *q*-value of less than 0.05 was considered statistically significant. Data were analyzed using SPSS 25.0, unless otherwise specified. Plots were generated by means of STAMP software and GraphPad Prism version 8.0.2.

## 3. Results

### 3.1. Cross-Sectional Study and Clinical Assessments

There were not significant differences in the age and BMI between both groups. As expected, complete blood count parameters, hepatic, renal and coagulation markers exhibited significant differences among groups (Table 1). Unsurprisingly, scores for liver disease (AST/ALT ratio, APRI and FIB-4 index) and systemic inflammatory marker (neutrophil-to-lymphocyte ratio), were statistically different (*p* < 0.001). All inpatients were under lactulose and antibiotic treatment, and were allocated in the Child–Pugh C class. Hepatic encephalopathy (HE) was one of the most frequent complications within the cirrhotic inpatients group (88.9%); none of them was classified in West Haven grade IV.

### 3.2. Microbiota Diversity between Groups

**Figure 1:**
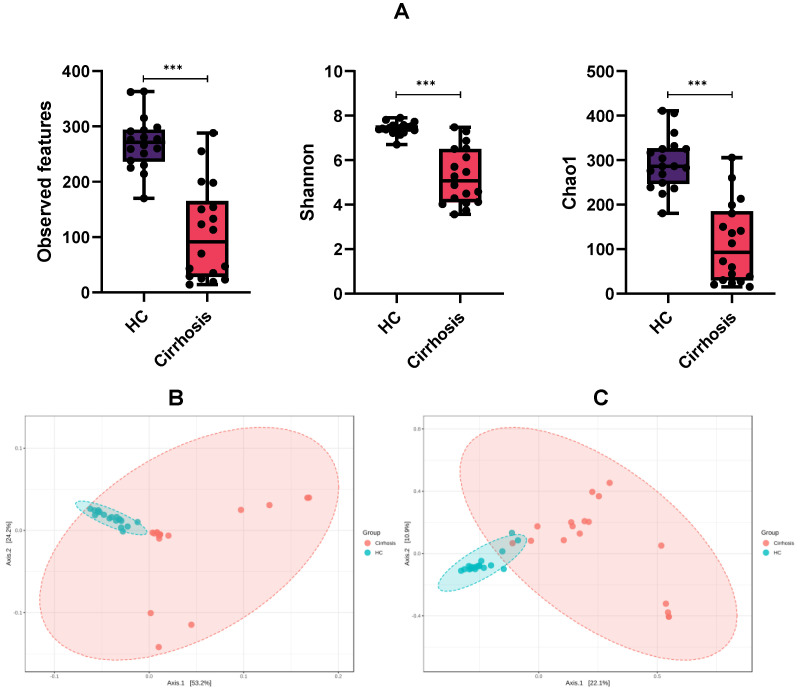
Microbiota richness and diversity. (**A**) Alpha diversity indices: Observed features, Shannon and Chao1 indices of healthy controls group compared with patients with cirrhosis. Mann-Whitney U test, *** *p* < 0.001; (**B**) Beta diversity plots for weighted and unweighted (**C**) UniFrac distances in cirrhosis group (red) and healthy control group (HC, blue). PERMANOVA tested, *p* < 0.001 in both cases.

Alpha diversities were calculated using observed features (analogous to observed ASVs), Shannon and Chao1 indices. Observed features express the species richness in a community; Shannon estimates richness and diversity, and Chao1 estimates diversity based on abundance (Figure 1A). We observed a significant decrease on the three indices within the cirrhosis group, compared with the healthy control group (HC) (*p* < 0.001). After controlling for other variables, such as upper gastrointestinal bleeding (UGIB), acute kidney injury (AKI), proton-pump inhibitor usage or infection at admission, no significant differences for alpha diversity were detected (Figure S1).

Beta diversity analysis was evaluated by weighted and unweighted UniFrac metrics, to show similarities or dissimilarities in bacterial diversity among the studied groups (Figure 1B,C). Results were plotted by Principal Coordinate Analysis (PCoA). We clearly observed the conformation of two well-defined groups, both in weighted UniFrac and unweighted UniFrac plots, which implies an evidently different microbiome profile among two groups (PERMANOVA, *p* < 0.001). As described above, once other variables were adjusted (UGIB, AKI, proton-pump inhibitor usage or infection), no significant differences were found among patients (Table S1) in regard to beta diversity.

### 3.3. Detected ASVs across Different Taxonomic Levels

**Figure 2:**
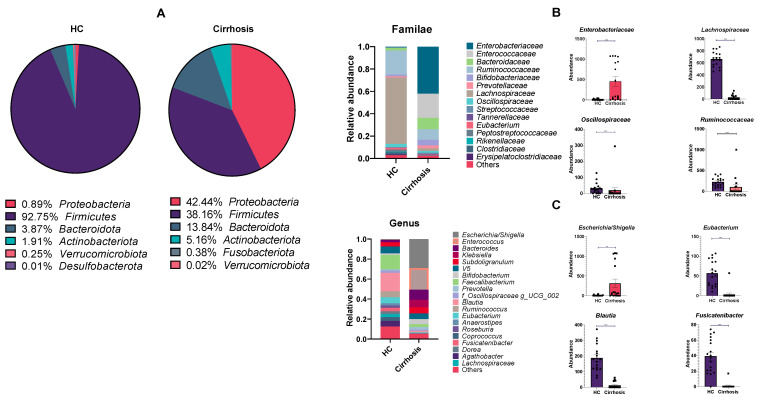
Relative abundance plots of healthy controls group (HC) and patients with cirrhosis group. (**A**) Pie chart representing different phyla in both groups. Phyla are accompanied by relative abundance value (%); (**B**) Stacked bar plot of bacterial familiae in HC group and cirrhosis group (**A**); on the right, increased abundance of Enterobacteriaceae in cirrhosis group are observed, while increased abundance of Lachnospiraceae, Oscillospiraceae and Ruminococcaceae are characteristic of HC group. Below (**C**), stacked bar plot of bacterial genera in HC group and cirrhosis group; in concordance with previous plot, on the right increased abundance of *Escherichia*/*Shigella* is characteristic of cirrhosis group, whereas *Eubacterium*, *Blautia* and *Fusicatenibacter* abundance exhibit a significantly increased abundance in HC group. Mann-Whitney U test, ** *p* < 0.01, *** *p* < 0.001.

Sequencing results showed an increased shift of bacterial populations at phylum level, with the top 5 most predominant phyla in the cirrhosis group compared with HC being Proteobacteria (42.44% vs. 0.89%), Firmicutes (38.16% vs. 92.75%), Bacteroidetes (13.84% vs. 3.87%), Actinobacteria (5.16% vs. 1.91%), and Fusobacteria (0.38% vs. 0%) (Figure 2A).

These results reflected a dramatic reduction in Firmicutes phyla, which encompasses physiologically relevant Gram-positive bacterial populations, at the expense of an increment of Proteobacteria in patients with cirrhosis.

At the family level, an Enterobacteriaceae and Enterococcaceae expansion was observed in the cirrhosis group, compared with the HC group (*p* < 0.001). Meanwhile, HC were marked by a marked abundance of Lachnospiraceae, Ruminococcaceae and Oscillospiraceae familiae (Figure 2B, *p* < 0.001), compared with the cirrhosis group.

Similar findings were reached at the genus level, where a profound increase of pro-inflammatory and pathogenic genera such as *Escherichia*/*Shigella* (*p* < 0.001), *Enterococcus*, *Bacteroides* and *Klebsiella*, characterized this group. We found that the HC group exhibited high abundance of genus *Blautia*, *Eubacterium*, *Fusicatenibacter*, *Roseburia* and *Faecalibacterium* (Figure 2C). Remarkably, these genera are known as SCFA producing bacteria, which were observed to be depleted in the cirrhosis group.

### 3.4. Linear Discriminant Analysis Effect Size (LEfSe)

**Figure 3:**
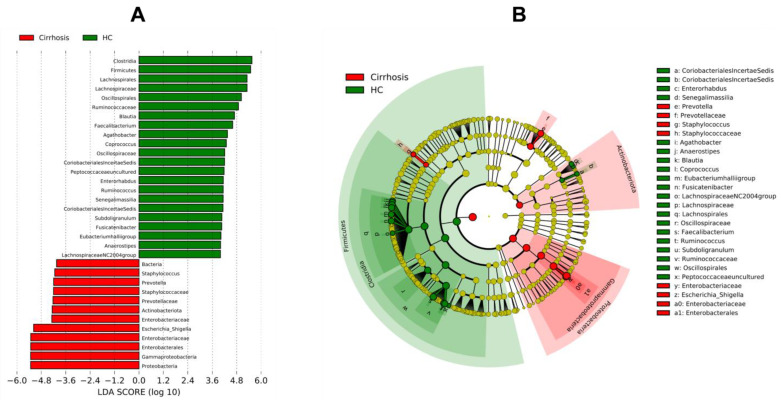
LEfSe analysis identifying key taxa in intestinal microbiota from patients with cirrhosis versus healthy control group (HC). (**A**) Bar plot showing LDA scores (Log > 4; *p* < 0.05); (**B**) Cladogram showing differentially abundant taxa at phylum, class, family, and genus levels between the two groups. Red circles indicate the remarkable taxa in the cirrhosis group while the green designates the HC group.

LEfSe was performed to determine key taxa between groups, across different taxonomic levels. This analysis revealed that the cirrhosis group was characterized by the predominance of bacteria to *Proteobacteria* phylum, such as *Escherichia*/*Shigella*. Other abundant key taxa within the cirrhosis group were phylum Actinobacteria, family Prevotellaceae and genus *Prevotella*, as well as *Staphylococcus*. On the other hand, the HC group was characterized by bacteria mainly belonging to phylum Firmicutes such as *Clostridia*, *Blautia*, *Faecalibacterium*, *Agathobacter*, *Ruminococcus* and *Fusicatenibacter* (Figure 3A,B). These findings are in concordance with relative abundance results (Figure 2B,C).

### 3.5. Functional Metagenomic Profiles

**Figure 4:**
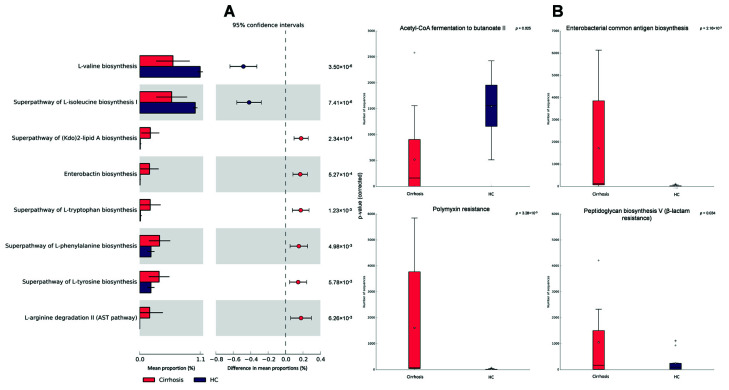
PICRUSt2 functional prediction of the patients with cirrhosis (red) compared with healthy controls (HC, purple). (**A**) Comparisons among MetaCyc pathways shown by mean proportion and difference in mean proportions; (**B**) Most relevant pathways are shown individually. The analysis was done with Welch’s inverted method, *p*-values were filtered with a cut-off value of *p* < 0.05.

PICRUSt2 analysis showed that pathways involved in the acetyl-CoA fermentation to butyrate (*p* < 0.05, Figure 4B) and biosynthesis of branched amino-acids (L-valine and L-isoleucine) are enriched in the HC group, compared with the cirrhosis group (*p* < 0.001, Figure 4A). Conversely, pathways related to the inflammation process (enterobacterial/LPS common antigen biosynthesis, *p* < 0.001), antibiotic resistance (polimyxin resistance [*p* < 0.001] and β-lactam resistance [*p* < 0.05]), as well as aromatic amino acids biosynthesis (L-tryptophan, L-phenylalanine, L-tyrosine, *p* < 0.001 in the three cases, Figure 4B), are significantly increased in the cirrhosis group. Interestingly, L-arginine degradation (AST pathway), which is an ammonia-producing pathway, is also increased in this group (*p* < 0.001).

### 3.6. Compositional Methods (ANCOM)

ANCOM (analysis of the composition of microbiomes) is a method which takes into account the compositional nature of microbiome data [41]. The result of this analysis is a volcano plot, where the statistically significant genus for each group is depicted (Figure S2). Accordant with previous analysis, we observed that *Escherichia*/*Shigella* is one of the most characteristic ge nera of patients with cirrhosis. Rather, *Coprococcus*, *Blautia*, *Agathobacter*, *Fusicatenibacter*, *Clostridia*, *Dorea*, *Ruminococcus*, *Eubacterium hallii* group, as well as Lachnospiraceae family, characterized the HC group.

### 3.7. Fecal Short-Chain Fatty Acid Concentrations

**Figure 5:**
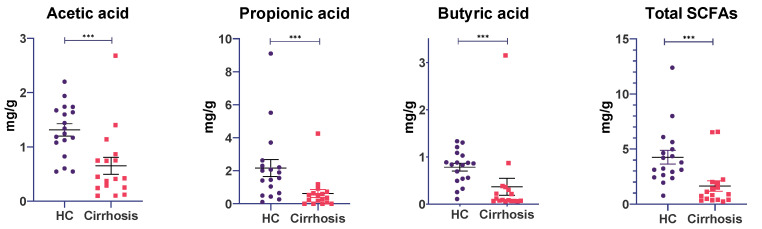
Short-chain fatty acids assessment in healthy controls (HC) and cirrhotic patients. The figure shows separate concentrations of acetic, propionic, butyric acids and total SCFAs. Results are expressed as mean ± SEM. Analyzed by Mann-Whitney U test, *** *p* < 0.001.

As expected from functional and differentially taxa analysis, SCFA concentrations in patients with cirrhosis were significantly lower, compared with healthy controls, including acetic, propionic and butyric acids (*p* < 0.001, Figure 5). Acetic acid was the most abundant SCFA in patients with cirrhosis, followed by propionic and butyric acid. In healthy controls, propionic acid was the most abundant SCFA, followed by acetic and butyric acid.

These measurements show the impaired production of physiologically important SCFA, which strongly correlate to the depletion of SCFAs producing genera in the cirrhosis group (*Faecalibacterium*, *Roseburia*, *Blautia*, *Agathobacter*, *Ruminococcus*).

## 4. Discussion

Globally, alcohol accounts for 30–50% of the mortality of cirrhosis-related causes [44]. Despite the intestinal microbiota’s important role in the pathophysiology of cirrhosis and its complications [2,7,45,46,47], there is scarce information on its alterations in the mestizo-Mexican population, and even less on the levels of SCFA in these patients.

In this pioneering study, we describe the alterations in the intestinal microbiota in western Mexican patients with decompensated alcohol cirrhosis. Specifically, we demonstrate disturbances in the gut intestinal microbiota diversity, and its functional impact on SCFA production. We also identify the characteristic taxa in patients with cirrhosis, as well as the metabolic pathways prediction; all of these being valuable findings not yet described in the global literature.

We unveil interesting findings regarding the alpha diversity, where total abundance and richness were significantly diminished in patients with cirrhosis, compared with the healthy control group; this being in line with the study of Bajaj et al., also in the Mexican population [4]. Additionally, we reported a vastly divergent beta diversity between both studied groups, reflecting taxonomic profiles with well-differentiated characteristics. Previous reports have found that differences in beta diversity can be associated with the complications of decompensated cirrhosis, such as infections or acute-on-chronic liver failure (ACLF) [48]. Regarding the taxonomic profile of the patients with cirrhosis, we observed a dramatic decrease in the Firmicutes phylum compared to the healthy control group, this at the expense of a shocking increase in the Proteobacteria phylum. The marked polarization of Proteobacteria with a relative abundance of 42.44% has not been described in patients with decompensated cirrhosis; the reported values are 15.8% [49]. Moreover, we observed a discrete increase in the Bacteroidetes phylum in this group. Interestingly, at the genus level, the most abundant taxon was *Escherichia*/*Shigella*. To find differentially abundant taxa in the studied groups, we used the LEfSe analysis and, as a different analytical approach, ANCOM. Both approaches confirmed the genus *Escherichia*/*Shigella* in the cirrhosis group.

*E*. *coli*, along with other species from the Enterobacteriaceae family, is frequently localized in the ascitic fluid of patients with cirrhosis; its presence significantly increases the probability of complications and raises mortality [6,50]. It is essential to highlight that *E*. *coli* is the specie that most frequently translocates from the intestinal lumen to the systemic circulation [6]. Furthermore, the lipopolysaccharides (LPS) of this species have a greater immunogenic potential than those of other enterobacteria [51]. On the other hand, in the healthy group, SCFA-producing bacteria and 7α-dehydroxylation bacteria were identified; these include *Blautia*, *Agathobacter*, *Fusicatenibacter*, and *Ruminococcus*, among others [52].

Continuing with the LEfSe analysis, the cirrhosis group was also represented by *Prevotella* and *Staphylococcus*, which belong to the Bacteroidetes and Firmicutes phylum, respectively. *Staphylococcus* is a commensal genus that can be found on the oral microbiota [53]. It has been suggested that in patients with cirrhosis, the alterations in the oral-gut-liver axis favor the trespass of commensal bacteria from the oral cavity to the gastrointestinal tract, this due to the immune alterations in the oral epithelium, the dysregulation in the bile acid conversion, and the concomitant use of proton pump inhibitors [7,54]. We also found *Prevotella* as a significantly enriched taxon in the cirrhosis group. This genus is considered an immunogenic commensal that, in a healthy population, is part of the SCFA producer enterotypes [55]. Paradoxically, in cirrhosis patients, the relative abundance of *Prevotella* is increased [5,45]. In this context, this genus has been related with a low-grade systemic inflammation, which is a crucial factor in developing more severe scenarios in decompensated cirrhosis, such as ACLF [56].

Interestingly, the relative abundance of other commensal bacteria in the healthy population, like *Bacteroides*, also increased in the group with cirrhosis. It is relevant to observe that *Prevotella* and *Bacteroides* have structurally analogous LPS, capable of inducing a similar inflammatory response [57,58].

The significant decrease in fecal SCFA in our study is synchronized with the loss of taxa belonging to the Firmicutes phylum, such as *Blautia*, *Eubacterium*, *Ruminococcus*, or *Fusicatenibacter*. These taxa are important SCFA producers and bile acid conversion modulators, and possess critical anti-inflammatory properties [52,59,60,61]. Although it has been previously demonstrated that there is a reduction in SCFA-producing bacteria in decompensated cirrhosis (mainly butyrate-producers) [19], this study describes, for the first time, a significant decrease in acetyl-CoA to butyrate fermentation in the cirrhosis group. The previous finding was described by the metabolic pathway prediction analysis. This parameter is in line with the significant decrease in fecal butyrate concentration quantified by gas chromatography. These findings strengthen the present study on the possible implications of the loss of this metabolite in the patient population of western Mexico, which is described below.

Physiologic hypoxia in the colon is mainly achieved through β-oxidation of butyrate by the colonocytes [62,63,64]. However, during gut dysbiosis, as well as in the absence or decrease of butyrate, colonocytes reduce their oxidative capacity and favor anaerobic glycolysis, resulting in a greater lactate production and oxygen availability in the intestinal lumen. As a consequence, there is an increase in the availability of alternative electron acceptors [64]. It is noteworthy that potential beneficial bacteria, such as *Roseburia*, *Blautia*, *Agathobacter*, and *Ruminococcus*, are strict anaerobes, contrary to those belonging to the Enterobacteriaceae family, which are facultative anaerobes [52,59,65,66]. Therefore, we believe that this change in the energy metabolism, which favors the oxygen in the mucosa, impacts the marked dysbiosis observed in the patients with cirrhosis described in the present study.

An additional mechanism that shows the relationship between the expansion of the genus from the Enterobacteriaceae family and the inflammatory state, as well as the increase in the colon and mucosal oxygenation, is the use of nitrate and nitrite (NO_x_) by this family. The NO_x_ are subproducts of nitric oxide, produced in massive quantities as part of the inflammatory response. These subproducts are key in the development of a hyperdynamic circulation, which is a characteristic of decompensation in patients with cirrhosis [67,68,69]. The notion that the inflammatory milieu rich in these components favors, in a selective mode, the expansion of enterobacteria, comes from the fact that *E*. *coli* can utilize NO_x_ as electron acceptors, taking advantage of other species and increasing their abundance [66].

Considering this, our research group has recently demonstrated that the supplementation with butyrate exerts beneficial effects on the intestinal epithelium, positively regulating tight junction proteins, mitigating the immune response, and limiting the loss of bacterial diversity in the cholestasis experimental model [18].

Concerning the decrease in propionic acid observed in the cirrhosis group, it has been demonstrated that the physiological levels of this SCFA positively regulate the expression of specific lectines against Gram-positive bacteria, which contributes to the prevention of bacterial translocation [70].

Consistent with the great abundance of Enterobacteriaceae, the metabolic pathway showed a strong synthesis activity of the enterobacterial common antigen (ECA), a characteristic surface molecule of this family, in addition to the synthesis of enterobactin, an iron-transporting siderophore [71,72].

Interestingly, metabolic pathways related to polymixin and β-lactam resistance stand out significantly in the cirrhosis group. Patients with decompensated cirrhosis have a high risk of severe bacterial infection; therefore, prophylaxis with third-generation cephalosporins is recommended [73]. Nevertheless, the prevalence of β-lactam resistance infections has increased in parallel with the frequency and lethality [74,75,76]. Moreover, empiric antibiotic prophylaxis, added to the multiple hospital admissions, is a factor that results in the gain of antibiotic resistance genes in the intestinal microbiota [77,78]. Finally, it is worth mentioning that the results of this prediction analysis have been scantly described in patients with decompensated cirrhosis.

Additionally, the alterations inferred by PICRUSt2 in the bacterial metabolism of amino acids showed an increase in the synthesis of aromatic amino acids (AAA) and ammonia-generating pathways, compared to a decrease in the synthesis of branched-chain amino acids (BCAA). This increase might be related to the elevation in the molar ratio of AAA/BCAA detected in the plasma of patients with cirrhosis [79,80]. Although the measurement of these metabolites was not the central objective of this study, the metabolic disruptions of the intestinal microbiota could be paramount in the development of frequent complications in our group of patients, such as hepatic encephalopathy. Hence, we consider it is relevant to prospectively measure these metabolites in our study population. It is worth mentioning that these pathways have been scarcely documented during the progression of alcoholic cirrhosis.

One hypothesis that explains the AAA/BCAA unbalance implicates that hepatic dysfunction elevates blood ammonia levels; therefore, as a compensation mechanism, there is a diminished BCAA storage in the skeletal muscle to allow ammonia catabolism at the expense of protein degradation [79]. This scenario is exacerbated under situations of systemic inflammation, as it frequently occurs in patients with cirrhosis [81]. Consequently, AAA have a more significant influx through the blood-brain barrier, disrupting the synthesis of neurotransmitters and giving place to “false” neurotransmitters, such as phenylethanolamine and octopamine [79]. These possible mechanisms are relevant in our study population, among which (88.9%) of the patients have or have had hepatic encephalopathy. Likewise, we are interested in assessing “false” neurotransmitters in a prospective study.

On the other hand, in our study all the patients were under antibiotic and lactulose treatments; therefore, it was not possible to validate the effect of these two variables on the alpha and beta diversity of the microbiota. However, previous studies in patients with different etiology and at various stages of cirrhosis, including ACLF, strongly suggest that the effects of antibiotics and lactulose are minimal or null in the composition and diversity of the intestinal microbiota [82,83,84,85]. Similarly, the inclusion of typical alterations of decompensated cirrhosis in this study, such as upper gastrointestinal bleeding, acute renal failure, IBP use, or the infection upon admission, did not impact the significant differences in the microbiota alpha and beta diversity (Supplementary Figure S1 and Table S1). The patients with decompensated cirrhosis included in this study reflect their actual status, where the microbiota alterations seem to be more related to the evolution of the disease itself than to possible confounding factors, such as pharmacological treatments.

Finally, we describe some of the limitations of our study. First, based on its transversal nature, it is impossible to infer the causality of the described phenomena. Furthermore, it was a single-center study with a relatively low number of patients. Additionally, due to sex differences in hospitalized patients with alcoholic cirrhosis, we only included male sex patients; thus, the results cannot be generalized to females. Another possible limitation is the lack of an extensive dietary evaluation, which was not carried out on the participants. Moreover, the study group was made up of Mexican patients from the same region; hence, the nature of the diet prevents the extension of these results to patients with decompensated cirrhosis that have a different dietary profile.

## 5. Conclusions

Our results show that the intestinal microbiota of patients with decompensated alcohol cirrhosis is dominated by the family Enterobacteriaceae and the genus *Escherichia*/*Shigella*. Additionally, this pathogenic and pro-inflammatory taxonomic profile correlates with a significant decrease in bacterial diversity, and a depletion of SCFA-producing bacteria with anti-inflammatory roles. In parallel, we observed a significant decrease in the fecal concentration of SCFA, which coincided with the metabolic pathway prediction, strongly suggesting that the fermentation of acetyl-CoA to butyrate is abolished in this group of patients. Likewise, metabolic pathways related to polymyxin and β-lactam antibiotic resistance, as well as those related to aromatic amino acids biosynthesis, are substantially disrupted. These metabolic pathways, occurring during the progression of alcoholic cirrhosis, have been poorly documented. Overall, these findings help improve our understanding of the pathophysiological bases of the gut-liver axis within the context of decompensated alcohol-related cirrhosis. This work is the pioneer for patients from western Mexico. Further studies with more patients are required to confirm our findings. 

## Figures and Tables

**Table 1:** Demographic and clinical characteristics of participants.

Characteristics	Healthy Controls (*n* = 18)	Cirrhosis (*n* = 18)	*p* Value
Mean age (years)	48.72 ± 8.63	49.89 ± 11.49	0.733 ^a^
BMI (kg/m^2^)	25.85 ± 2.86	24.66 ± 3.82	0.355 ^b^
Hemoglobin (g/dL)	15.1 ± 0.9	8.66 ± 2.4	0.000 ^a^
Platelets (10^9^/µL)	229.59 ± 63.5	140.32 ± 90.18	<0.002 ^a^
White blood cells (10^9^/µL)	5.68 ± 1.42	17.13 ± 14.23	0.000 ^b^
Neutrophils (10^9^/µL)	3.29 ± 1.05	14.01 ± 12.92	0.000 ^b^
Lymphocytes (10^9^/µL)	1.8 ± 0.43	1.53 ± 0.94	<0.001 ^b^
Total bilirubin (mg/dL)	0.67 ± 0.17	7.72 ± 8.99	0.000 ^b^
Direct bilirubin (mg/dL)	0.13 ± 0.05	2.26 ± 3.74	<0.002 ^b^
GGT (IU/L)	25.67 ± 13.07	116.22 ± 92.83	0.000 ^b^
Albumin (g/dL)	4.47 ± 0.32	2.1 ± 0.58	0.000 ^b^
ALT (U/L)	26.94 ± 12.91	31 ± 17.07	0.284 ^a^
AST (IU/L)	22.89 ± 9.23	79.06 ± 46.98	0.000 ^b^
ALP (IU/L)	69.67 ± 22.21	142.39 ± 59.78	0.000 ^b^
Total protein (g/dL)	7.16 ± 0.47	2.94 ± 1.35	0.000 ^b^
Creatinine (mg/dL)	0.82 ± 0.12	1.42 ± 0.86	<0.008 ^b^
Prothrombin time (s)	11.49 ± 0.77	23.26 ± 8.55	0.000 ^b^
INR	1.09 ± 0.08	2.13 ± 0.79	0.000 ^b^
Sodium (mmol/L)	N/A	131.94 ± 6.46	N/A
Child–Pugh score	N/A	11.39 ± 1.33	N/A
MELD-Na score	N/A	28.11 ± 6.69	N/A
Prior or actual HE	N/A	16 (88.9)	N/A
West Haven grade (1/2/3/4)	N/A	1/8/7/0	N/A
Ascites	N/A	14 (77.8)	N/A
Upper gastrointestinal bleeding (UGIB)	N/A	6 (33.3)	N/A
Acute kidney injury	N/A	5 (27.8)	N/A
Lactulose	N/A	18 (100)	N/A
Mean arterial pressure (mmHg)	N/A	78.06 ± 12.05	N/A
Duration of antibiotic treatment (days)	N/A	3.94 ± 4.15	N/A
Antibiotic type ^c^ (*n*)	0	18	N/A
Only ceftriaxone	-	4	
Ceftriaxone + rifaximin	-	5	
Ceftriaxone + other	-	3	
Others	-	6	
Use of proton pump inhibitors	0 (0)	8 (44.4)	N/A
Infection at admission	N/A	10 (55.6)	N/A
Neutrophil-to-lymphocyte ratio	1.87 ± 0.58	10.01 ± 10.46	0.000 ^b^
AST/ALT ratio	0.93 ± 0.26	5.89 ± 1.57	0.000 ^b^
APRI index	0.22 ± 0.13	1.49 ± 1.16	0.000 ^b^
FIB-4 index	1.1 ± 0.66	6.55 ± 4.04	0.000 ^b^

Abbreviations: N/A (not assessed), BMI (body mass index), HE (hepatic encephalopathy), MELD-Na (Model for End-stage Liver Disease-Sodium), GGT (gamma-glutamyltransferase), AST (aspartate aminotransferase), ALT (alanine aminotransferase), ALP (alkaline phosphatase), INR (international normalized ratio), APRI (AST to Platelet Ratio Index). Data are expressed as means ± standard deviation or number of patients (%). ^a^ Student’s *t*-test, ^b^ Mann-Whitney U test. ^c^ Antibiotic treatment was categorized in 4 groups: only ceftriaxone, ceftriaxone plus rifaximin, ceftriaxone plus other, and others (clarithromycin, piperacillin/tazobactam, linezolid, metronidazole, levofloxacin and ciprofloxacin). Patients could receive more than one antibiotic.

## Data Availability

Data is contained within the article.

## Supplementary Materials

The following supporting information can be downloaded at: https://www.mdpi.com/article/10.3390/microorganisms10061231/s1, Figure S1: Alpha diversity metrics between healthy controls (HC) and subgroups of cirrhotic patients, Figure S2: Volcano plot depiction of ANCOM analysis, Table S1: Statistical significance in beta diversity metrics between healthy controls (HC) and subgroups of cirrhotic patients.
