# Supplementary material for: Escherichia/Shigella, SCFAs, and Metabolic Pathways—The Triad That Orchestrates Intestinal Dysbiosis in Patients with Decompensated Alcoholic Cirrhosis from Western Mexico
Source: Microorganisms. 2022 Jun 16;10(6):1231. doi: 10.3390/microorganisms10061231 (PMC9229093; doi:10.3390/microorganisms10061231)
Supplement: Supplementary file 1 [file microorganisms-10-01231-s001.zip › microorganisms-1745206-supplementary.pdf]

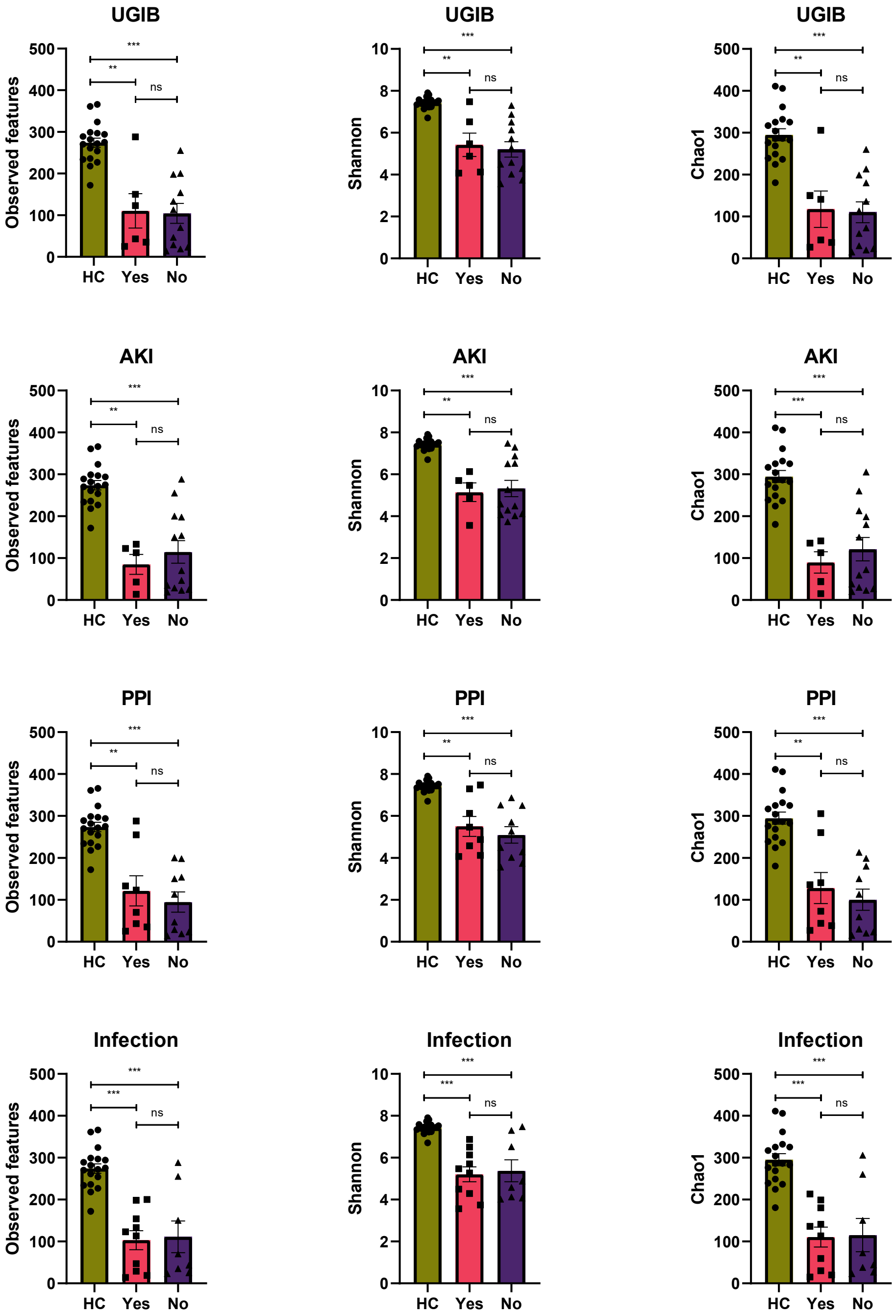

Supplemental Figure S1: Alpha diversity metrics between healthy controls (HC) and subgroups of cirrhotic patients. The figure shows alpha diversity indices for patients subgrouped by clinical settings: UGIB (upper gastrointestinal bleeding), AKI (acute kidney injury), PPI (proton pump inhibitors usage) or infection at admission. No significant differences were found among patients subgroups. Results are expressed as mean ± SEM. Analyzed by Kruskal-Wallis test plus Benjamini, Krieger and Yekutieli method for multiple comparisons. ** p<0.01, *** p<0.001, *ns*: not significant.

Supplemental Table S1: Statistical significance in beta diversity metrics between healthy controls (HC) and subgroups of cirrhotic patients.

Pairwise PERMANOVA results with Benjamini-Hochberg multiple testing corrections				
**Weighted UniFrac distance**				
Group 1	Group 2	Pseudo-F	*p*-value	*q*-value
**UGIB**				
HC	No	4.00684379	0.001	0.0015
HC	Yes	8.16378484	0.001	0.0015
No	Yes	0.53754516	0.789	0.789
**AKI**				
HC	No	5.09231	0.001	0.0015
HC	Yes	10.925914	0.001	0.0015
No	Yes	2.012356	0.083	0.083
**PPI usage**				
HC	No	4.874918	0.001	0.0015
HC	Yes	5.53117	0.001	0.0015
No	Yes	0.396942	0.919	0.919
**Infection**				
HC	No	5.07362	0.001	0.0015
HC	Yes	5.397498	0.001	0.0015
No	Yes	0.449855	0.879	0.879
**Unweighted UniFrac distance**				
Group 1	Group 2	Pseudo-F	*p*-value	*q*-value
**UGIB**				
HC	No	5.51644845	0.001	0.0015
HC	Yes	3.88655291	0.001	0.0015
No	Yes	0.64289723	0.891	0.891
**AKI**				
HC	No	5.43499453	0.001	0.0015
HC	Yes	4.2303456	0.001	0.0015
No	Yes	0.92991691	0.415	0.415
**PPI usage**				
HC	No	5.57567668	0.001	0.0015
HC	Yes	4.25779142	0.001	0.0015
No	Yes	0.91627568	0.447	0.447
**Infection**				
HC	No	4.73186942	0.001	0.0015
HC	Yes	4.7676101	0.001	0.0015
No	Yes	0.54556854	0.982	0.982

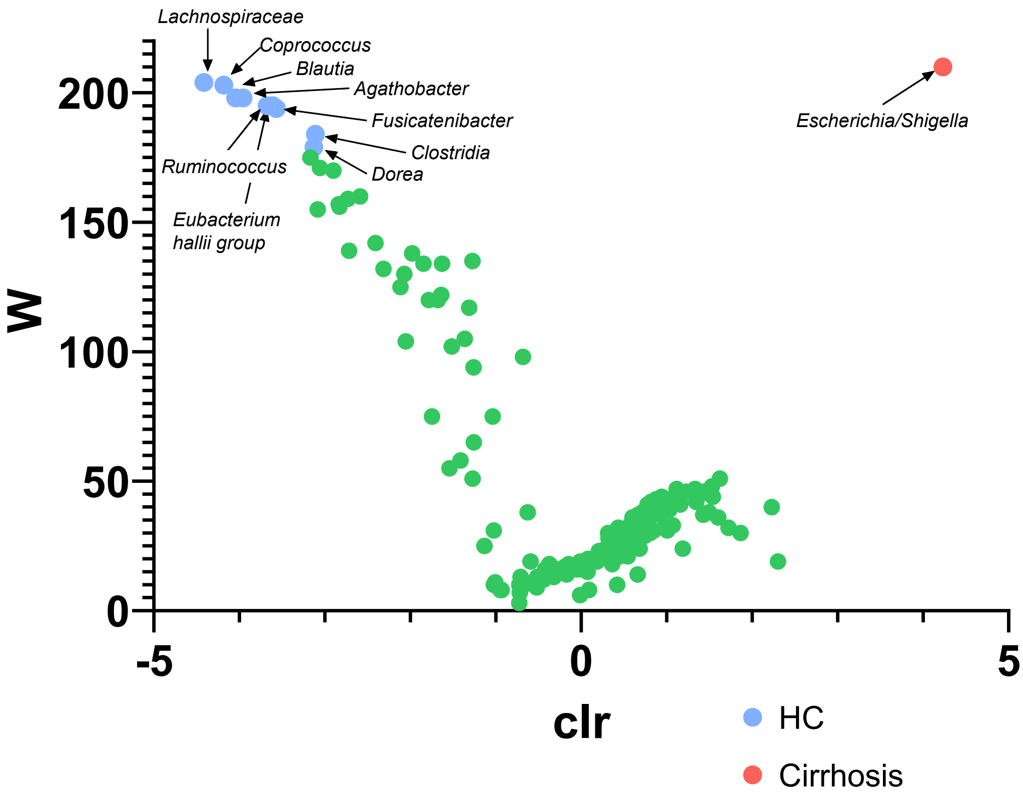

Supplemental Figure S2: Volcano plot depiction of ANCOM analysis. The horizontal axis is the centered log ratio transformation (clr) representative of the difference in abundance of a significant taxonomical unit between healthy controls (HC, blue dots) and patients with cirrhotic (red dots). A green color indicates an overlap of ASVs between groups. The W statistic indicates the value of the statistical test corresponding to the number of times the null hypothesis was rejected for each taxonomical class.
